# Lessons Learned From Cases of COVID-19 Infection in South Korea

**DOI:** 10.1017/dmp.2020.141

**Published:** 2020-05-07

**Authors:** Yun-Jung Kang

**Affiliations:** Department of Clinical Laboratory Science, Sang-ji University, Wonju, Korea

**Keywords:** COVID-19, large groups, mass infection, mask, prevent, sanitary glove

## Abstract

On December 31, 2019, the Chinese government officially announced that the country had a single pneumonia case with an unknown cause. In the weeks after, South Korea had 24 confirmed cases by February 8, and the number has increased steadily since then. The highly contagious virus known as coronavirus disease 2019 (COVID-19) infected Case No. 31 in Daegu; she was the first patient related to Sincheonji Church. Later, the number of cases involved with Sincheonji skyrocketed. On March 6, 2020, the number of confirmed cases was 6284, with 42 dead. This study, through collecting epidemiological data about various COVID-19 infection cases, discovered that getting together in large groups leads to mass infection, and that paying close attention to personal hygiene by means of wearing masks, sanitary gloves, etc., can prevent the spread of COVID-19. Additional epidemiological data and related studies on COVID-19 infections in South Korea are likely to support or slightly modify this conclusion. However, this study is significant in that it emphasizes the precautionary principle in preventing and managing infectious diseases, and has a suggestion for public health policies, which are currently in high demand.

On December 31, 2019, the Chinese government announced officially that the country had a pneumonia case with an unknown cause. It is not clear when this case occurred initially. According to reports containing 41 confirmed cases from the medical teams in Wuhan, China (December 1, 2019 to January 2, 2020), the first patient developed symptoms on December 1, but had never visited the Huanan fish market.^1^ Later, on January 7, 2020, the Chinese government reported a new kind of coronavirus as the cause of this pneumonia case and provided information on the virus to researchers around the world.^2^ The World Health Organization (WHO) named the virus coronavirus disease 2019 (COVID-19) temporarily, but as the English name is rather long in Korean, the South Korean government decided to call it “Corona 19 (Corona il-gu),” following the opinion of the Korean Centers for Disease Control and Prevention.^3^

Coronavirus (CoV) is a virus that can infect humans and various other animals. It is an RNA virus with gene sizes from 27 to 32 kb, with four genera (alpha, beta, gamma, and delta). Alpha and beta genera can infect humans and animals, while gamma and delta genera infect only animals other than human beings. As implied by its name, the shape of the virus observed through an electron microscope is a crown shape with characteristic protein spikes attached to a ball. The pathogen is COVID-19, and the source of infection is estimated to be an animal, although the investigation is ongoing. The infection route channel is estimated to be animal → human → human. Spread between humans is assumed to be due to droplet infection. Secondary infection cases in households and hospitals have been confirmed. Clinical manifestations are fever, respiratory symptoms (coughs and dyspnea), and pneumonia.^4^

The first death from COVID-19 was reported on January 10 in China. On January 13, 2020, Thailand reported the first case confirmed outside of China’s borders. The patient never visited the fish market in Wuhan. After that, Japan reported the first case in its territory on January 15, 2020. South Korea followed suit on January 20. On February 8, the confirmed cases in South Korea reached 24, and the number of patients is increasing to this day (Table 1).^5^ COVID-19, with is strong infectivity, infected Case No. 31 in Daegu. The Korea Centers for Disease Control and Prevention reported Case No. 31 of COVID-19 at 10:00 am, February 18, 2020. A female in her 60s living in Daegu, Case No. 31 was found to be admitted to a hospital at the time. She attended a service at Sincheonji Church in Namgu, Daegu, on February 9 and 16, 2020, each for 2 h.^6^ After this first confirmed case related to the Sincheonji Church in Daegu (Case No. 31), the number of COVID-19 cases by Sincheonji members in South Korea increased sharply. The South Korean government, using the church member registry of 244,743 members it acquired from Sincheonji headquarters, analyzed the connection between church members and 4212 COVID-19 cases confirmed until March 2. According to the analysis, 93% of the confirmed cases were related to Sincheonji.^7^ The possibility of mass infection in church services was suggested. As more than 6000 COVID-19 cases were confirmed, the cases of infection became more diverse.

This study collected and studied various cases of COVID-19 infections to the time of this writing to determine measures of prevention against the spread of the disease, while suggesting a managerial direction for public health, which is in urgent demand now.

## METHODS

### Data Collection

Reports were collected from South Korea based on data from the Korea Centers for Disease Control and Prevention, along with data and presentation examples from the Korea Centers for Disease Control and Prevention from February 8 to March 6, 2020.

## EXPLANATION OF TERMS

### Definition of COVID-19

Respiratory syndrome caused by severe acute respiratory syndrome coronavirus 2 (SARS-CoV-2) infection.

### Classification of Diseases

Forensic Infectious Disease: Class 1 Infectious Disease, New Infectious Disease Syndrome.

### Diagnostic Criteria

#### Patient

A person who has been confirmed to be infected with an infectious pathogen according to the examination criteria for diagnosis.

Test criteria for diagnosis: Virus isolation from samples, detection of specific genes in samples.

### The Korea Centers for Disease Control and Prevention

The Korea Centers for Disease Control and Prevention is an organization belonging to the Ministry of Health and Welfare of Korea and is located in Cheongju City, North Chungcheong Province. This institution is responsible for the prevention, investigation, quarantine, testing, research, and long-term transmission management of infectious diseases, chronic diseases, rare intractable diseases, and injured diseases to improve the national health of South Korea.

### Large-Scale (Group) Infection

The term *large-scale group infection* refers to a condition in which a large number of people are exposed to each other in a short period of time, because some infectious diseases are clustered among schools, companies, dormitories, factories, or people in a limited physical space at once, and the conditions for causing infectious diseases are established as a group.

### Sporadic Infection

Some disorders occur sporadically in time or place. It is not so important in public sanitation, but it is sometimes necessary to monitor the situation as it happens as an example of group outbreak.

## RESULTS

### Occurrence Types of COVID-19: Mass Infections

#### Sincheonji Church in Daegu

Nationally, 71.7% of the confirmed COVID-19 cases in Korea are verified to be related to mass infections. The other 28.3% cases are due to sporadic infections or the causes are still being investigated. Investigating the mass infection cases by regions, confirmed that cases related to Sincheonji Church in Daegu accounted for 72.4% of the overall 3397 confirmed cases in the city. More cases were confirmed in public and medical facilities while investigating people who had had contact with the Sincheonji followers.^8^

#### Oncheon Church

In Busan and Gyeongnam, the number of COVID-19 confirmed cases related to a retreat to Oncheon Church is 35. People from Busan account for most of the cases, with the number of 33; the other two people were from Gyeongnam. Among the 35 confirmed cases, 28 were members of the congregation, and they have infected 7 others who came into contact with them. The Oncheon Church case occupies 35.5% of the total confirmed cases in Busan, 93 being the city’s total number of patients.^9^

#### Geochang Church

Geochang Church bore the most COVID-19 confirmed cases as a single group in a single region of Gyeongnam province. Therefore, Gyeongnam province government sent an epidemiological investigation team to Geochang and started an in-depth investigation in cooperation with the city government of Geochang. The number of the members of Geochang Church congregation is 60, with 39 people living in Geochang and 21 coming from other towns. Among the 39 members from Geochang, 10 people were confirmed to be infected, while the other 29 were confirmed negative. Sixty-two people who had contacted the confirmed cases are all confined in their own homes.^10^

#### Pilgrims to Israel

Among the 39 pilgrims of Andong, Gyeongbuk parish of the Catholic Church, who had participated in the pilgrimage to Israel, 30 people were confirmed to be infected by COVID-19. Nineteen other people who had contacted with the confirmed cases were also tested to be positive, making 49 the total number of the confirmed cases. As some of the pilgrims are reported to have visited restaurants and eateries after returning to Korea, there is a possibility of regional spread.^11^

#### Saengmyeongsam Church in Suwon

The mass infection among the Saengmyeongsam Church congregation, which bore 10 confirmed cases of COVID-19, is reported to have been caused by worshipping and eating together in a small and crowded indoor environment. A Sincheonji believer and lecturer from Sincheonji Church, Daegu, who attended the service, is indicated to be the index case.^12^

#### Daenam Hospital in Cheongdo

A total of 118 COVID-19 cases were confirmed among the patients and medical personnel of Daenam Hospital in Cheongdo, Gyeongbuk. This is the second largest mass infection case, following the Sincheonji Church case. As many as seven people died. Among the confirmed cases other than the dead, 101 of the 103 inpatients of the psychiatric ward were infected, leaving only 2 patients unaffected.^13^

#### St. Mary’s Hospital in Eunpyeong-gu, Seoul

The number of COVID-19 confirmed cases related to St. Mary’s Hospital in Eunpyeong-gu, Seoul, increased to 14. The quarantine officials regard this case as the biggest mass infection in Seoul and are continuing the investigation. Among the 14 confirmed cases, 4 are inpatients, 5 are family members of the patients, 2 are caregivers, 1 is a transferring agent, and 2 are others (a care worker and a blood-donation bus worker). Classifying according to their residences, 6 live in Eunpyeong-gu, 2 in Gangdong-gu, 2 in Jongro-gu, 2 in Seodaemun-gu, 1 in Yangcheon-gu, and 1 in Goyang-si.^14^

#### Hanmaeum Hospital in Changwon

Hanmaeum Hospital in Changwon, where medical personnel were confirmed positive of COVID-19 subsequently, was the first place quarantined as a cohort. The total number of the confirmed cases was 7. On February 26, 2020, the provincial government of Gyeongsangsnam-do announced that it had decided to quarantine the hospital as a cohort for 14 days after consulting with the Korea Centers for Disease Control and Prevention, recognizing the seriousness of this situation. The number of people cohort-quarantined is 100, including 91 inpatients and medical personnel.^15^

#### Jaesaeng Hospital in Bundang

A mass infection occurred among medical personnel and inpatients in Jaesaeng Hospital in Bundang, Sengnam-si in Gyeonggi-do. A total of eight cases, including two nurses, three nurses’ aides, and three patients, were confirmed to be positive in COVID-19 diagnostic checks.^16^

#### Mil-al Sarang-eu-Jib, a Facility for People with Serious Handicaps in Chilgok

On February 25, 2020, a total of 22 confirmed cases of COVID-19 occurred in Mil-al Sarang-eu-Jib, a facility for people with serious handicaps in Gasan-myeon, Chilgok-gun in Gyeongbuk. According to the public health authorities and the provincial government of Gyeongsangbuk-do, 21 people at the facility were confirmed to be infected by COVID-19, including 11 inpatients, 5 staff, and 5 challenged workers. With another case confirmed the day before, the total number of cases here is 22.^17^

#### Seorin Sanitarium in Gyeongsan

Another mass infection case occurred at the Seorin sanitarium. with 13 confirmed cases of COVID-19. The first patient is reported to have come in contact with a Sincheonji Church member.^18^

#### Pureun Sanitarium in Bongwha, Gyeongbuk

Forty-nine confirmed cases of COVID-19 were reported at the Pureun Sanitarium: 39 inpatients, 9 care workers, and 1 nurse’s aide. A total of 116 people live in or work for the Pureun Sanitarium, with 56 inpatients, 42 in-house staff, and 18 workers of the day care center. The proportion of confirmed cases reach 42.2% among the in-house staff.^19^

#### Zumba Dance Studios

The second confirmed case in Cheonan, Chungnam, a zumba dancer in her 50s, gave lessons to others in three separate studios in the city. Later, 80 cases were confirmed from seven sports facilities in Chungnam, with zumba dance studios in Cheonan at the center. Among them, 4 are instructors, 50 are attendees, and 26 are family members and other contacts.^20^

#### An Apartment Building in Seongdong-gu

Thirteen cases of COVID-19 occurred in an apartment building with stores on the ground floor in Seongdong-gu, Seoul. The route channel of infection spread is thought to be the residents of the building → a member of the managerial staff → his family members → colleagues of the family members.^21^

#### A Private Educational Institute

As a series of COVID-19 infection cases are occurring in a private educational institute in Busan, the Busan Office of Education (with superintendent of education Seokjun Kim) took measures, including sending text messages to all parents of students attending kindergartens, elementary schools, middle schools, and high schools, pleading with them not to send their children to private educational institutes until the spread of COVID-19 moderates. Currently, four cases were confirmed (including two students) from a private educational institute in Jingu, Busan.^22^

#### A Coin Karaoke in Changnyeong

During the recent week, as many as six confirmed cases of COVID-19 infection were traced to a coin Karaoke establishment in Changnyeong-eup, Changnyeong-gun, Gyeongnam. They are Cases No. 51 (61, female), 56 (30, male), 61 (30, male), 70 (24, male), 71 (16, female), and 76 (24, male). Case No. 51 is the manager of the coin karaoke, while four among the others (Case Nos. 61, 70, 71, and 76) were visitors to the place.^23^

### The Occurrence Types of COVID-19: Sporadic Infections

#### Home

Among the COVID-19 confirmed cases in Korea, two were confirmed tertiary infection cases following secondary infection, spreading social anxiety. Person A, Case No. 6 (55), was confirmed to have been infected after eating together with Person B, Case No. 3 (54), in a restaurant (Hanil-gwan) in Gangnam-gu, Seoul. Person A infected two of his family members, leading to the first tertiary infection cases in Korea. Until now, the number of contacts with Person A is suspected to be 8, including his family members.^24^

#### Wedding Hall

Person A, a confirmed case who is currently being treated in a management ward, reported that he was infected after visiting a wedding hall in Daegu. He spent only 1 h and 10 min in the wedding hall. On the date of visiting, his wife and son wore masks, unlike himself.^25^

#### Elevator

Person A (53, male), a curate of Myeongseong Church in Gangdong-gu, and Person B (41, female), a resident of the same apartment building he lived in, took the same elevator, and both were confirmed positive. Person B did not wear a mask in the elevator and became infected with COVD-19 after the short time spent in an elevator.^26^

#### Internet Café

In Busan, a middle school student (teenager) was confirmed to be infected with COVID-19 on February 28, 2020, after visiting the same Internet café where a member of the Oncheon Church had visited.^27^

### Cases of Preventing the Spread of COVID-19

#### Daily Record of COVID-19

Person A, a tour guide, confined himself in his own home when he first developed symptoms (a sore throat) on January 31, 2020. Since then, he recorded his COVID-19 symptoms meticulously in a daily journal. When going to hospitals, he avoided using public transportation and walked, taking only uncrowded routes. He wore masks and gloves even in his home, which led to the negative confirmation of 23 people who had come in contact with him, including his mother living with him under the same roof.^28^

#### Home Confinement

Patient number 63 from Bukgu, Busan, who is in his 30s, confined himself to his home when he heard that he was confirmed positive of COVID-19 and drove by himself even when he visited the selective care center. He always wore masks in his own home and separated his living area, saving his parents; they were both confirmed negative.^28^

#### Masks and Sanitary Gloves

A COVID-19 confirmed Case C (58, male) wore masks and sanitary gloves even in his homes when he developed symptoms of the virus. He sterilized the dishes he used in hot water right after using. Even though he was confirmed negative once, he recorded his symptoms and the places he went just in case. After he was confirmed, he provided this information to the prevention authorities and contributed to their quick reaction.^29^

Pyeongtaek city government in Gyeonggi-do announced that all the family members of the second COVID-19 confirmed case were confirmed negative of the virus on February 24, 2020. CCTV footages showed that the patient always wore the mask.^30^

## DISCUSSION

**FIGURE 1:**
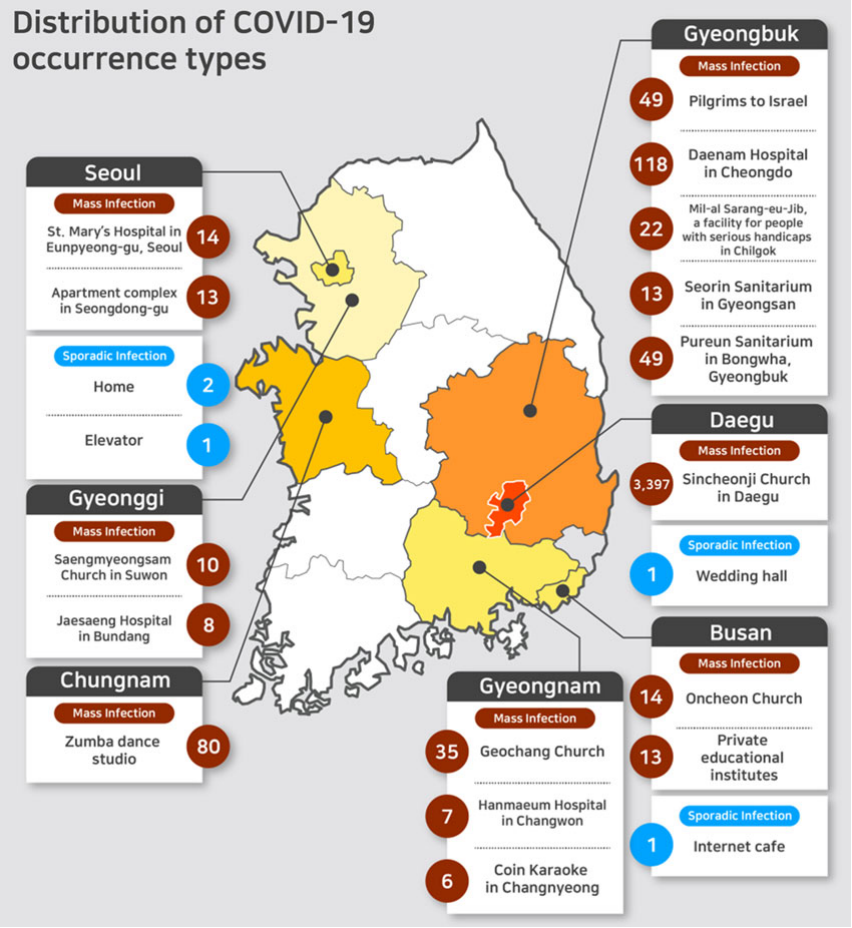
Distribution of COVID-19 Occurrence Types.

According to the Korea Centers for Disease Control and Prevention, 71.7% of COVID-19 cases in South Korea are related to mass infections. The other 28.3% of the cases were related to sporadic infections or are still being investigated as to the cause.^8^ This study agrees in that most cases are related to mass infections (Table 2; Figure 1). Locations of mass infections are churches, hospitals, sanitariums, private educational institutes, and dance studios, where many people gather in a closed and crowded environment and tend to contact each other at a close distance. The Chinese National Hygiene and Health Committee admitted the possibility of infection through aerosols in its 6th edition of COVID-19 Treatment Guide, providing “long-time exposures to dense aerosol in relatively closed environment.”^31^ Aerosol means solid or liquid small particles floating in the air, ranging from 0.001 μm to 100 μm.^32^ According to scientific definitions, small particles that can travel far distances for a long time are classified as airborne, while bigger aerosols are classified as droplets.^33^

**TABLE 1:** Present Condition of COVID-19 Occurrences in Korea (00:00, March 6^th^)

Category	Sum	Present Confirmed Case Conditions				Present Test Conditions		
		Sum	Lifted Quarantine	Quarantined	Deaths	Sum	Tested	Confirmed Negative
00:00 03/05	146,541	5,766	88	5,643	35	140,775	21,810	118,965
00:00 03/06	164,740	6,284	108	6,134	42	158,456	21,832	136,624
Fluctuations	+18,199	+518	+20	+491	+7	+17,681	+22	+17,659

**TABLE 2:** Distribution of COVID-19 Occurrence Types

Occurrence Type	Region	Details	Number of Confirmed Cases
Mass infection	Seoul	St. Mary’s Hospital in Eunpyeong-gu, Seoul	14
		Apartment complex in Seongdong-gu	13
	Busan	Oncheon Church	35
		Private educational institutes	4
	Daegu	Sincheonji Church in Daegu	3,397
	Gyeonggi	Saengmyeongsam Church in Suwon	10
		Jaesaeng Hospital in Bundang	8
	Chungnam	Zumba dance studio	80
	Gyeongbuk	Pilgrims to Israel	49
		Daenam Hospital in Cheongdo	118
		Mil-al Sarang-eu-Jib, a facility for people with serious handicaps in Chilgok	22
		Seorin Sanitarium in Gyeongsan	13
		Pureun Sanitarium in Bongwha, Gyeongbuk	49
	Gyeongnam	Geochang Church	35
		Hanmaeum Hospital in Changwon	7
		Coin Karaoke in Changnyeong	6
Sporadic infection	Seoul	Home	2
		Elevator	1
	Daegu	Wedding hall	1
	Busan	Internet café	1

**TABLE 3:** Cases of Preventing the Spread of COVID-19

Prevention Type	Details	Prevention Effect
Daily record of COVID-19	Home Confinement COVID-19 logbook showing symptoms and movements Wearing a mask and sanitary gloves at home	All contacts 23 negative result
Home confinement	Home Confinement Wearing a mask even at home Separating family and living space	Parents negative result
Masks and sanitary gloves	Wearing a mask and sanitary gloves at home Disinfect your own dishes in boiling water COVID-19 logbook showing symptoms and movements	All family negative result

This means that the spread through aerosols can manifest in two types, one being droplet infection by direct contact and one being airborne spread. Generally, droplet infection occurs when the infected person sneezes, coughs, talks, or exhales; this is called the first aerosolization.^34^ In contrast, airborne spread is due to the spread of droplet nuclei with the size of <5 μm, left behind when the water in droplets evaporates. As the droplet nuclei are light and can float in the air for a long time, they can be especially dangerous.^35^

Although religious services conducted in crowded spaces like churches and temples are vulnerable to infections, there are no specific prevention guides from the government. Most churches hosted services as usual, despite the possibility of regional infection with more and more cases of COVID-19 cases being confirmed. Contact between hundreds and thousands of the members in the same space was inevitable, making people vulnerable to COVID-19 with its strong infectivity. Furthermore, the Korea Centers for Disease Control and Prevention suggests the possibility of limited but constant spread in groups through holiday services and small meetings.^36^ Private educational institutes and physical training facilities bear similar dangers. Even though the government postponed the start of semesters for kindergartens, elementary schools, middle schools, high schools, and universities for fear of mass infections, many private educational institutes and physical training centers are insisting on operation, threatening COVID-19 prevention.

According to a report on March 6, 2020, by the provincial government of Gyeonggi-do and Gyeonggi Office of Education, only 9932 among 33,091 private educational and training facilities closed on March 4, with a close rate of 30%. This means that 23,159 facilities, or 70% of the total institutes, are still running. Due to their noncompliance with the strong government measures postponing the start of semesters, the threat of COVID-19 infection and spread is increasing dramatically. The Office of Education cannot force private institutes to cease operation as there is no legal basis. Voluntary cooperation is desperately needed.^37^

There are even cases of infection that go against the explanation provided in the Chinese National Hygiene and Health Committee’s 6th edition COVID-19 Treatment Guide, which says that the condition for spread through aerosol is “long-time exposures to dense aerosol in relatively closed environment.”^32^ The cases of infection in the elevator and the wedding hall, where the patients spent only a short amount of time together, prove this false. The patients of these two cases are similar in that they did not wear masks. From the perspective of prevention and management, blocking the route of airborne infection is critical.

There were also many desirable cases where the infection spread was prevented by using masks and sanitary gloves (Table 3).^28-30^ Patients, after feeling their symptoms or finding out that they had contact with confirmed cases, confined themselves to their homes and shunned crowded places to prevent infecting others. They always wore masks and sanitary gloves, and even though their uninfected family members were under the same roof, these measures allowed them to be confirmed negative. The mask used at this time was a disposable non–woven-material mask. In contrast, when the confirmed cases did not wear masks or confined themselves in separate rooms in their homes, all their family members were diagnosed to be positive.^24^ These are cases where wearing masks and using proper individual hygiene decided the positive outcome of prevention.

The temporary conclusion of this study, based on limited epidemiological data and information on confirmed cases currently available, is that group meetings lead to mass infections of COVID-19, and that caring for individual hygiene by wearing masks and sanitary gloves can prevent its spread. However, in the current epidemic, the group that needs the most masks are the medical staff working on the front lines. Therefore, an individual’s wearing of a mask should be limited to patients suspected of having an infection. This conclusion can be supported or modified according to additional epidemiological data and research results on COVID-19 infections in South Korea. However, this study is meaningful in that it emphasizes the precautionary principle in preventing and managing infectious diseases, and has a suggestion for public health, which is currently in urgent demand.

## Availability of data and materials

The datasets used and/or analyzed during the current study are available from the corresponding author on reasonable request.
